# Olfactory misinformation provides refuge to palatable plants from mammalian browsing

**DOI:** 10.1038/s41559-024-02330-x

**Published:** 2024-02-02

**Authors:** Patrick B. Finnerty, Malcolm Possell, Peter B. Banks, Cristian Gabriel Orlando, Catherine J. Price, Adrian M. Shrader, Clare McArthur

**Affiliations:** 1 School of Life and Environmental Sciences, University of Sydney, Sydney, NSW Australia; 2 Mammal Research Institute, Department of Zoology and Entomology, University of Pretoria, Pretoria, South Africa

**Keywords:** Behavioural ecology, Conservation biology

## Abstract

Mammalian herbivores browse palatable plants of ecological and economical value. Undesirable neighbours can reduce browsing to these plants by providing ‘associational refuge’, but they can also compete for resources. Here we recreated the informative odour emitted by undesirable plants. We then tested whether this odour could act as virtual neighbours, providing browsing refuge to palatable eucalyptus tree seedlings. We found that protection using this method was equivalent to protection provided by real plants. Palatable seedlings were 17–20 times more likely to be eaten by herbivores without virtual, or real, neighbours. Because many herbivores use plant odour to forage, virtual neighbours could provide a useful practical management approach to help protect valued plants.

## Abstract (web summary)

Unpalatable plants can deter herbivory on palatable plants grown in the same vicinity, but managing unwanted herbivory by planting unpalatable plants is not time- or cost-effective. This paper shows that artificial odour, designed to mimic the informative volatile compounds of unpalatable plants, can deter herbivory on the target plants, offering a potentially useful management strategy

## Main

Foraging decisions of mammalian herbivores can have costly consequences, devastating areas of habitat restoration and post fire recovery^1,2^, and cause billions of dollars of damage in forestry and agriculture^3^ globally. Current solutions to problematic herbivory traditionally target removing animals, such as lethal control, or preventing access to plants, such as fencing. These approaches are costly and increasingly limited by practicalities, concerns over animal welfare and non-target ecological effects, so alternative approaches are needed. The most effective alternatives are likely to be those based on understanding and harnessing foraging cues, motivations and decisions^4^ of the herbivores.

Generalist mammalian herbivores typically forage by navigating heterogenous landscapes of discontinuous food resources. To maximize foraging efficiency in such landscapes, animals seek high-quality food patches and avoid low-quality patches dominated by unpalatable low-nutrient, chemically defended^5,6^ or physically obstructing^7,8^ plant species. Palatable plants in such low-quality patches can receive protection against herbivores from their low-quality neighbours—termed associational plant refuge^9,10^.

To recognize and select among plants and plant patches, many mammalian herbivores use and rely on plant odour^11,12^. But plant odours are extremely complex. They often comprise hundreds of volatile organic compounds (VOCs), many of which are common among plant species^13^ and most likely to be uninformative noise. The VOC information that foraging mammalian herbivores use to recognize plants amidst this complex olfactory noise remains poorly understood.

Defining odour information is crucial to understanding its role in plant–herbivore interactions, in foraging and more broadly in any ecological interactions mediated by odour. It could also be crucial for developing new ways to manage problem browsing^14^. For example, strategically designed artificial odours could be exploited to inform herbivores, altering their foraging decisions and nudging them away from valued plants. Deploying informative odours in place of real plants sends a deceptive message and hence is a form of olfactory misinformation^14,15^.

**Fig. 1:**
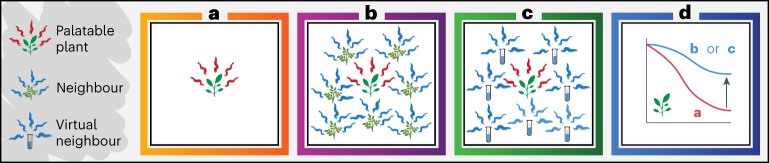
Conceptual model using patch-scale informative odour to protect palatable plants. **a**, A palatable plant emitting odour providing a cue to foraging herbivores. **b**, A real avoided, low-quality plant neighbourhood provides associational refuge, protecting the palatable plant from herbivores by degrading actual patch quality and delaying browsing at a patch. **c**, A virtual neighbourhood of artificial informative odours mimicking real avoided neighbours replaces real plants yet still protects the palatable plant via associational refuge. **d**, Thus, population-level survival of **a** palatable plants is improved by **b** and **c** because many mammalian herbivores detect, identify and decide whether to visit and browse at food patches using odour.

Our aim (Fig. 1) was to test the use of artificial informative odours, acting as virtual neighbours in a patch, to degrade perceived patch quality and alter herbivore foraging decisions. Specifically, we tested whether informative VOCs of an unpalatable (low-quality) plant species could replace real plants yet still provide associational refuge to a palatable seedling of another species. We recently presented a novel practical approach to find and quantify informative VOCs based on two main criteria of reliability, consistency and precision^16^. In this Article, we use and test this approach for its effectiveness in defining informative VOCs.

As our model for the study, we used a free-ranging macropod, the swamp wallaby (*Wallabia bicolor*), foraging in eucalypt woodland in eastern Australia. Swamp wallabies are native, abundant, mid-sized (13–17 kg) browser/mixed feeders, ecologically equivalent to many species of deer, antelope and elephant in Europe, North America, Asia and Africa. Like these species, wallabies shape vegetative communities via selective browsing and are a known limiting factor to the recruitment and survival of plant species in regenerating or post-fire recovery areas^17,18^. Our unpalatable plant species was the pungent shrub, *Boronia pinnata*, and our palatable plant species was *Eucalyptus punctata*, a foundational canopy species. Both were part of the native vegetation open forest community.

**Extended Data Fig. 1:**
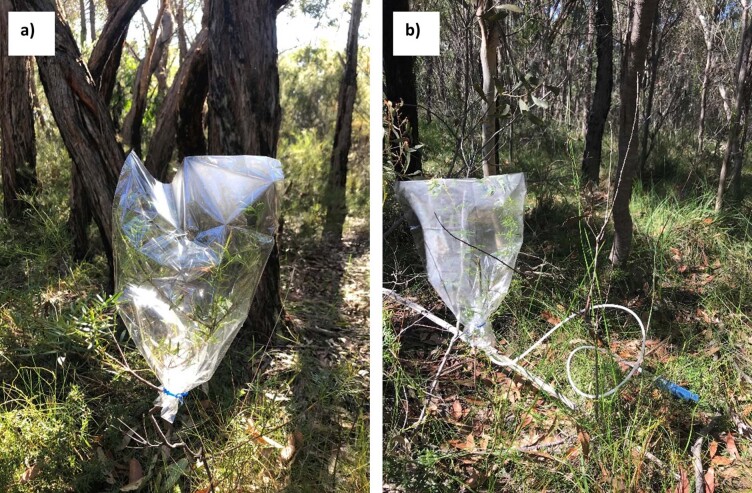
Odour ‘headspace’ sampling of B. pinnata. Odour ‘headspace’ sampling of B. pinnata for GC-MA analysis. **a)** Polyacetate oven bag over a single B. pinnata branch. Odour headspace was allowed to accumulate in the bag for 15 minutes prior to sampling. **b)** Air was extracted from the bag for 15 minutes through a thermal desorption tube filled with 200 mg of Tenax TA using a PAS500 Personal Air Sampler.

**Extended Data Fig. 2:**
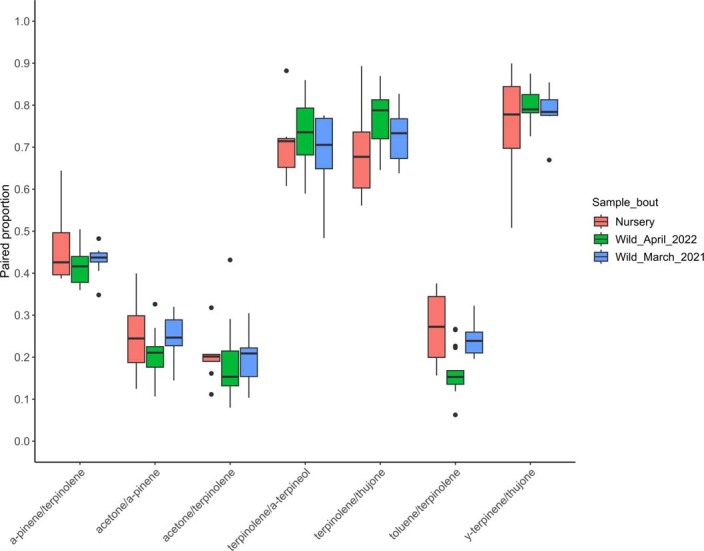
Paired VOC proportions across *B. pinnata* odour sampling bouts. Boxplots of paired VOC proportions sampled for all seven selected informative VOC pairs, across two sampling bouts for odour headspace of wild B. pinnata plants (March 2021, n = 10, April 2022, n = 20) and from branch enclosure sampling of nursery raised B. pinnata (n = 8). Plots represent the proportion of the first listed compound in each VOC pair (calculated after fourth-root transformation, see Methods section). Boxplots indicate the median, the first and third quartiles, and the maximum and minimum values. Closed circles indicate outliers.

**Extended Data Fig. 3:**
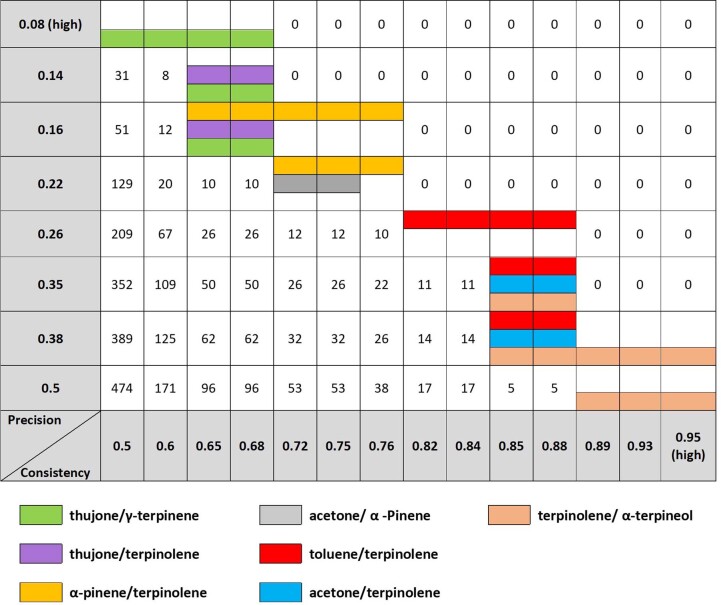
Informative *B. pinnata* VOC consistency-precision reliability space. Informative pairs of volatile organic compounds (VOCs) identified from B. pinnata presented in a consistency-precision reliability space. Here, values for the two ‘Rules of reliability’ are present on a scale of consistency (threshold baseline 0.5 to high consistency 0.95) and precision (threshold baseline 0.5 to high precision 0.08). Specific VOC pairs, presented in a unique colour, are shown when one to three pairs were detected. Where a cell contains the number 0, no pairs were identified. Where more than three pairs were identified, the number of pairs is shown.

To define the odour profile and determine putative informative VOC combinations for *B. pinnata*, we first collected ‘headspace’ VOC emissions from 30 plants across two sampling bouts (Extended Data Fig. 1 and Supplementary Note 1). We analysed these emissions using gas chromatography–mass spectrometry (GC-MS), producing the complete odour profile of 482 VOCs (see Supplementary data 1 and Extended Data Fig. 2 for a comparison of the odour profiles between sampling bouts). As many plant species emit VOCs in common, such VOCs may only convey information about a particular plant species if produced consistently and in distinct combinations for that species^16^, usually described in terms of VOC pairs^19^. To identify putatively informative VOC pairs, we used two ‘rules of reliability’^16^: VOC pairs needed to be emitted (a) consistently (by more than 50% of plants sampled) and (b) in precise proportions (between 0.5 (moderate precision) to 0 (absolute precision)). From these rules, we selected seven VOCs from a band of putatively informative VOC pairs (Extended Data Fig. 3): thujone, γ-terpinene, toluene, α-pinene, terpinolene, acetone and α-terpineol.

We then created three artificial odour treatments to act as virtual neighbours: informative, uninformative and flipped proportion. The informative treatment combined the seven VOCs (in six pairs) in correct informative proportions. The uninformative treatment combined seven new VOCs (in six pairs) that were recorded in *B. pinnata* but fell below our chosen reliability threshold. The flipped proportion treatment inverted the relative amount of informative VOCs within pairs (Extended Data Table 1). This treatment allowed us to test our prediction that the relative amounts of informative VOCs mattered, not simply their presence.

**Extended Data Fig. 4:**
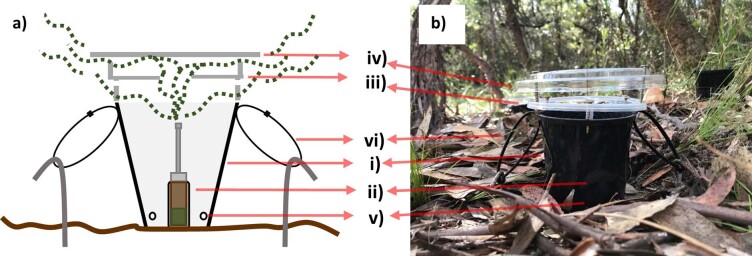
Virtual neighbour odour dispenser. **a)** A longitudinal schematic of a virtual neighbour odour dispenser (i) a black plastic 200 mm ‘Garden City Plastic Grow Plant Pot’ with a (ii) 2 cm piece of 19 mm black poly pipe inserted inside to securely fit a single vial. 5 mm above the open pot, using screws we attached a (iii) 220 mm round clear plastic lid with a 50 mm hole cut centrally above the vial to allow the odour from vials to escape. We placed (iv) an intact, larger 300 mm round clear plastic lid 5 mm above the first lid as a secondary rain guard. (v) Three small holes were drilled into the base of the pots to act as a flue to produce an air draft to help disperse odour from vials. (vi) Two 300 × 4.8 mm black cable ties were attached to the sides of each pot, which acted as an anchor point for tent pegs which were used to secure dispensers to the ground. Odour being emitted from vials is depicted as dashed green lines. **b)** A virtual neighbour odour dispenser attached to the ground in-situ at our study site.

**Extended Data Fig. 5:**
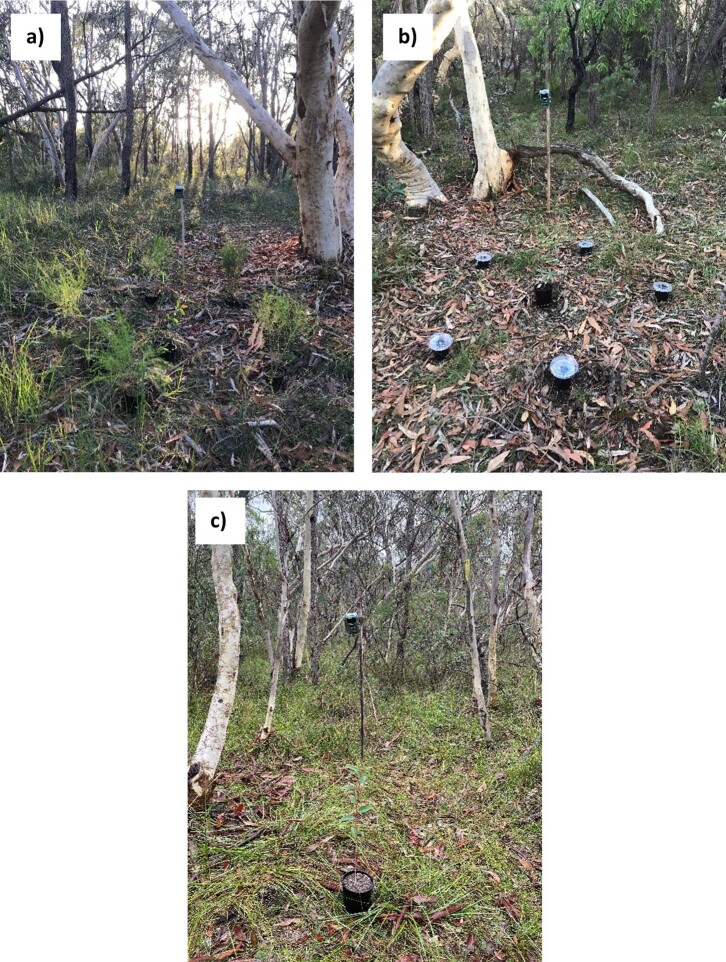
Associational refuge main trial treatments. Associational refuge main trial treatments **a)** real neighbourhood treatment of a single E. punctata surrounded by five B. pinnata, **b)** virtual neighbourhood manipulation treatment of a single E. punctata surrounded by five virtual neighbour odour dispensers, **c)** untreated control, a single E. punctata on its own, against background vegetation. Images show experimental set up of motioned-triggered cameras fastened to wooden stakes (camera height = 0.7 m, distance to seedling = 1.5 m) at an approximate 45° angel towards the focal seedlings.

To test how swamp wallabies responded to the three virtual neighbour treatments compared to real *B. pinnata*, we deployed them in the field with three additional treatments: real *B. pinnata*, a procedural control and an untreated control. The real *B. pinnata* treatment was a single *E. punctata* seedling surrounded by five evenly spaced *B. pinnata* plants, allowing a direct comparison between real and virtual neighbours. The untreated control treatment was a single *E. punctata* seedling. The procedural control treatment was a single *E. punctata* seedling surrounded by five empty virtual neighbour odour dispensers (Extended Data Figs. 4 and 5) to ensure that any wallaby browsing effects were not due to the presence of the dispensers themselves.

**Extended Data Fig. 6:**
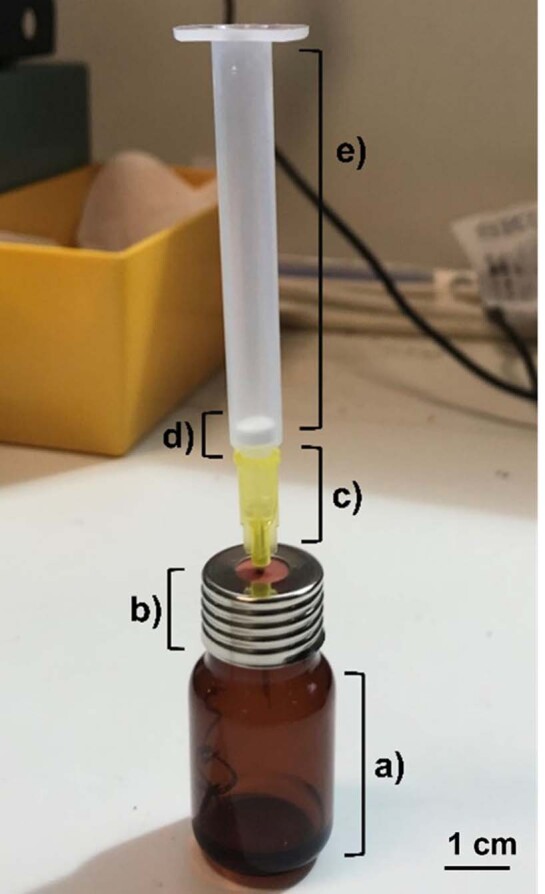
A virtual neighbour vial. A virtual neighbour vial comprising 2.96 ml VOC compound mixture in a (a) 10 mL glass amber vial, sealed with (b) PTFE lined butyl septa headspace cap. Vials are pierced with a diffusion tube made from a (c) 20 gauge syringe needle attached to a (e) polypropylene SPE tube containing a (d) 20μ porosity PE frit to limit the diffusion rate.

All six treatments were deployed at our study site in plots (*n* = 15 per treatment, at least 50 m apart) in a completely randomized plot design. At each plot, five virtual or real neighbours were placed in a circle (radius 1 m) around a single *E. punctata* seedling at the centre of the patch (Extended Data Fig. 5). For the virtual neighbours, artificial odour (made up to a total of 2.96 ml) was deployed in a glass amber diffusion vial (design based on ref. ^20^; Extended Data Fig. 6) placed in a custom-built odour dispenser (to protect them from rain (Extended Data Fig. 4)). We had confirmed that the VOC emission rate from 2.96 ml for the informative treatment was equivalent to the VOC emission rate of a single real *B. pinnata* shrub. We had also confirmed that the proportional change in emissions from the virtual neighbour treatments stayed constant over time for at least 60 days, indicating a steady emission rate throughout the experiment. To assess the effectiveness of the treatments, we quantified time taken for a wallaby to first browse the *E. punctata* seedling in the plot.

**Fig. 2:**
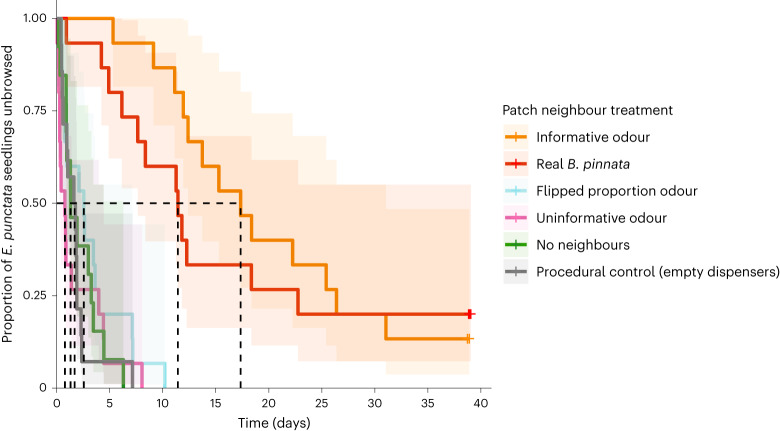
Survival curve (time to first browse) of palatable *E. punctata* seedlings as a function of patch neighbour treatment (*n* = 15 per treatment). The proportion of plots (±95% confidence intervals in shaded areas) remaining unbrowsed over 40 days. Cox proportional-hazard modelling showed a significant treatment effect (LR ${\chi}_{5}^{2}\,$ = 74.70, *P* < 0.0001). Dashed black lines indicate median survival time for each treatment.

Time to first wallaby browse on *E. punctata* seedlings differed significantly as a function of treatment (analysis of deviance likelihood ratio (LR) ${\chi}_{5}^{2}\,$ = 74.70, *P* < 0.0001; Fig. 2 and Extended Data Table 2). Informative virtual neighbours provided real browsing refuge from swamp wallabies, equivalent to protection provided by real *B. pinnata* plants (*P* = 0.72). Cox proportional hazard ratios indicated *E. punctata* seedlings were 20 times more likely to be browsed in the untreated control ‘no neighbours’ treatment than if surrounded by an informative virtual neighbourhood (*P* < 0.0001) and 17 times more likely to be browsed than if surrounded by a real neighbourhood of *B. pinnata* (*P* < 0.0001). *E. punctata* seedlings surrounded by flipped proportion and uninformative virtual neighbourhoods, as well as empty virtual neighbour vials (procedural control treatment), were equally likely to be browsed by wallabies as an *E. punctata* seedling ‘alone’ (Extended Data Table 2) (that is, no refuge effect). If *E. punctata* seedlings were browsed, wallabies generally ate all the foliage (75 of 82 seedlings) or most foliage (7 of 82 seedlings). Background wallaby activity did not differ across treatments (LR ${\chi}_{5}^{2}$ = 1.90, *P* = 0.86).

Our results show that informative virtual neighbourhoods provided associational browsing refuge to palatable seedlings, successfully replacing real neighbouring plants. We provide clear evidence that the specific subset of VOCs deployed in particular proportions designed to be informative were actually informative to wallabies and can be successfully deployed as olfactory misinformation to influence their foraging behaviour. Our results are a positive test for our approach^16^ to find and quantify informative VOCs within the complex odour profile of an individual plant species. That the flipped proportion treatment did not provide refuge confirms that the relative proportion of the informative VOCs matter, not just their presence.

Our findings provide an important step forward in improving our understanding of both fundamental and applied mammalian behavioural ecology, providing new insight into the ways in which mammalian herbivores detect and respond to the world around them. We argue that our approach to detect and quantify informative VOCs has the potential to be applied more broadly to develop targeted virtual plant neighbours specific to herbivores in other systems.

As a management tool to protect palatable seedlings, virtual neighbours offer many advantages over real plants. Real plants compete for water and resources, which can outweigh protective effects in providing browsing refuge^21^. With future development, virtual neighbours could also be deployed en masse, quickly and likely cheaply, last long term or be removed at will, and tweaked over time to avoid potential habituation.

Herbivore browsing damage varies in detail and context globally: different plants, different herbivores, different landscapes. However, irrespective of the context, the logical approach used to define the putatively informative compounds of plant species is likely transferable to many mammalian (or potentially invertebrate) herbivores that rely primarily on plant odour information to forage. Consequently, using similar olfactory misinformation tactics, virtual neighbourhoods represent a new approach that has the potential to be harnessed as a benign, non-lethal, cost-effective and novel tool for reducing problem herbivory in conservation (for example, threatened plant species) and management (for example, forestry and agriculture) globally.

## Methods

### Animal ethics statement

Animal ethics approval was granted by the University of Sydney’s Animal Ethics Committee (protocol number 2022/2196).

### Creating and deploying virtual neighbours

#### Odour profile of *B. pinnata*

To develop a complete odour profile of *B. pinnata*, we undertook ‘headspace’ VOC odour sampling of 30 naturally occurring *B. pinnata* plants across our study site in Ku-ring-gai Chase National Park, Sydney, Australia (33° 41′ 33″ S, 151° 08′ 44″ E) (Extended Data Fig. 1). Randomly selected individual plants sampled were of approximate equal height (198 ± 11 cm) and were at least 50 m away from any other sampled individual. Sampling was undertaken across two sample bouts (March 2021, *n* = 10, and April 2022, *n* = 20). Across both bouts, sampling was conducted between 9 a.m. and 4 p.m. Ambient temperatures recorded were similar across both bouts (March 2021, 20.8 °C to 24.3 °C; April 2022, 19.5 °C to 23.4 °C). Average daily rainfall was slightly higher in March 2021 (14.3 ± 4.7 mm) than in April 2022 (7.8 ± 3.0 mm).

To sample *B. pinnata* odour headspace, a polyacetate oven bag (Glad 35 cm × 48 cm) was placed over a single branch (branches used between individuals sampled were of approximate equal size, 6 × ‘biounits’ of 14 cm plant ‘lengths’). Headspace was allowed to accumulate in the bag for 15 min. Next, air was extracted from the bag for 15 min through a thermal desorption tube filled with 200 mg each of Tenax TA (Markes International) using a PAS500 Personal Air Sampler (Spectrex). All thermal desorption tubes were analysed within 2 weeks of sampling by desorbing samples with automated thermal desorption (ULTRA 2 and UNITY 2, Markes International) for 6 min at 300 °C and concentrated on a Tenax TA cold trap at −30 °C. This cold trap was then flash heated to 300 °C, and the concentrated sample injected splitless via a heated transfer line (150 °C) onto a 7890A GC-MS (Agilent Technologies) fitted with a BP1 capillary column (60 m × 0.32 mm, 1 µm film thickness; Agilent) at a flow rate of 2.3 ml min^−1^. The GC oven was heated at 35 °C for 5 min then 4 °C min^−1^ to 160 °C then 20 °C min^−1^ to 300 °C for 10 min. The GC was coupled to a mass-selective detector (Model 5975C; Agilent). The temperature of the GC-MS interface was 280 °C, the MS ion source 230 °C and the quadrupole 150 °C. The detector, in electron impact mode (70 eV), scanned the range of 35–300 *m*/*z*. Operation of the GC-MS was controlled by Agilent Chemstation (version E.02.01.117) and the ULTRA 2 and UNITY 2 by Maverick (version 5.0; Markes).

Common contaminating ions (73, 84, 147, 149, 207, 221 and 281 *m*/*z*) were removed from the chromatograms using the Denoising function in OpenChrom (version 1.1.0 (ref. ^22^)). Chromatograms were then processed using the MSeasyTkGUI package^23^ in RStudio (version 4.2.0, R Development Core Team 2022) and all putative compounds clustered. MSeasyTkGUI also produced peak areas for the putative compounds based on their total ion count (TIC). Blank samples (*n* = 7) were run in conjunction with all analysis; the upper 95% confidence interval of the mean blank value was subtracted from all samples. Final ion counts of the putative compounds emitted by our plants were obtained by subtraction of the background TIC recorded for each compound from the plant samples. Identification of putative compounds was made by a combination of manually comparing mass spectra against a commercial library (NIST14 library in NIST MS Search v.2.2f; NIST) and the library’s calculated match factor, using a threshold of 700. The final TIC of those putative compounds identified as the same compound was added up to obtain only one value per compound. In total, we identified 482 individual VOCs across all *B. pinnata* sampled from after blank subtraction (see Supplementary Note 1, Supplementary Data 1 and Extended Data Fig. 2 for a comparison of the odour profiles between sampling bouts).

#### Defining the informative VOCs of *B. pinnata* and developing virtual neighbour treatments

After using the two ‘rules of reliability’^16^, we selected seven VOCs from a band of putatively informative VOC pairs for *B. pinnata* (Extended Data Fig. 3) and combined them in appropriate proportions (based on average TICs for each VOC, from A1 above) to create (a) informative virtual neighbour treatment. To create (b) uninformative virtual neighbour treatment, we combined seven new VOCs and pairs that fell below our chosen reliability threshold: *d*-limonene, (1*R*)-(-)-myrtenal, nonanoic acid, β-myrcene, tridecane, 3-pentanone and geraniol, and to create (c) flipped proportion virtual neighbour treatment, we inverted the relative amount of informative VOCs within pairs (Extended Data Table 1).

We combined VOCs in the identified paired proportions so that the total volume of the mixture was 1 ml per treatment. Mixtures were measured into 10 ml glass amber virtual neighbour vials (Agilent) sealed with polytetrafluoroethylene-lined butyl septa headspace caps (Agilent). Vials were pierced with a diffusion tube made from a 20-gauge syringe needle (Sigma-Aldrich) attached to a polypropylene solid-phase extraction tube containing a 20 μm porosity polyethylene frit to limit the diffusion rate (based on ref. ^24^; Extended Data Fig. 6).

**Extended Data Fig. 7:**
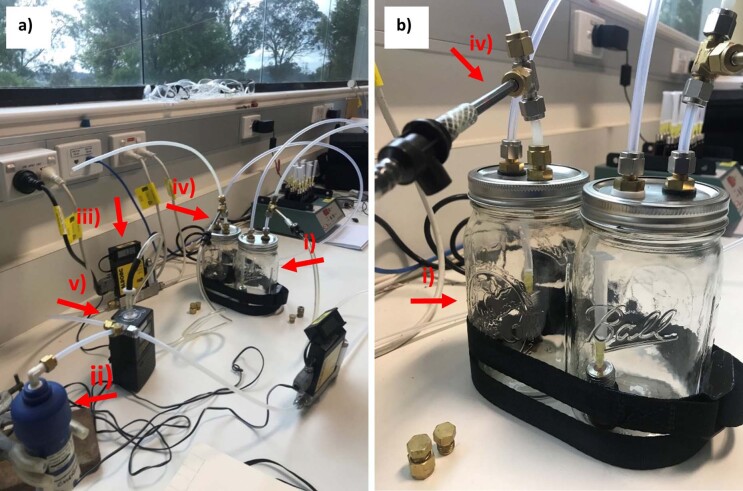
Virtual neighbour VOC sampling. a) Overview of virtual neighbour VOC sampling set-up. b) Virtual neighbours in mason jars undergoing VOC sampling. Virtual neighbour VOC sampling included (i) one litre glass Mason jars with lids fitted with ¼” brass bulkhead fittings to allow air in and out of the jar. Instrument air, passed through an activated charcoal scrubber (ii), was supplied to the Mason jar at 1.3 L min-1 using a mass flow controller (iii). VOCs were collected from the jar outlet using a sorbent tube containing 200 mg Tenax TA (iv) connected to an air pump (v) flowing air at 70 mL min-1 for 15 minutes.

As the GC-MS has different sensitivities to different compounds, we verified whether compounds mixed in virtual neighbour vials for each treatment matched the paired proportions from the *B. pinnata* plants. VOC samples from virtual neighbour vials (*n* = 10 per treatment) were measured dynamically using 1 l glass Mason jars (Ballmason Australia) where the jar lid was fitted with 1/4 inch brass bulkhead fittings (Swagelok) to allow air in and out of the jar (Extended Data Fig. 7). Instrument air (BOC), passed through an activated charcoal scrubber, was supplied to the Mason jar at 1.3 l min^−1^ using a mass flow controller (Aalborg). VOCs were collected from the jar outlet using a sorbent tube containing 200 mg Tenax TA (Markes International) connected to an air pump (AirChek 2000; SKC) flowing air at 70 ml min^−1^ for 15 min. Vials were allowed 15 min to acclimate to conditions within the jar before VOC sampling commenced. Background (control) samples were taken at the beginning and end of each day. Post-sampling tubes were maintained at 4 °C until analysis by GC-MS. Compounds were identified after GC-MS analysis using the same protocol used when measuring the odour profiles from *B. pinnata* plants. Emission rates (µg h^−1^) of each of the compounds were determined using their background subtracted concentrations, the chamber flow rate and the sampling duration. We adjusted compound volumes in the virtual neighbour treatments so their emissions matched those from the real plants.

#### Comparing emission rates of virtual neighbours to real *B. pinnata* neighbours

In our manipulative experiment, we deployed *B. pinnata* plants sourced from a nursery (Plants Plus Cumberland Forest Nursery, West Pennant Hills, Sydney) as neighbours. Therefore, we next compared and again adjusted our virtual neighbour odours so the relative proportions of VOC emissions and the absolute emission rates matched those of these plants (*n* = 8; height 650 ± 12 mm, biomass 45.7 ± 2.5 g above ground dry weight, calculated after odour sampling).

**Extended Data Fig. 8:**
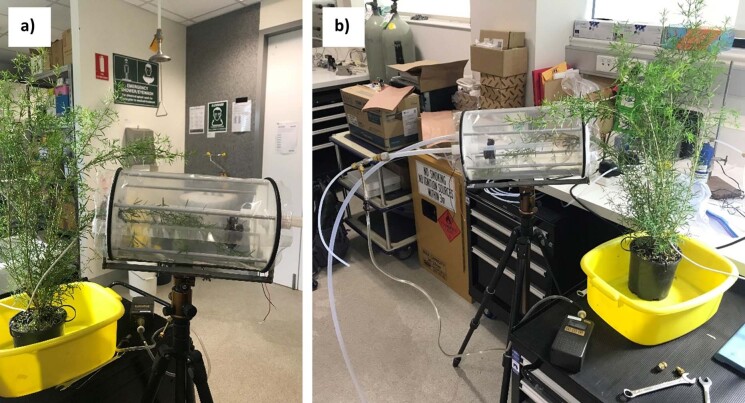
Sampling B. pinnata VOC emission rate. a) Here, a single branch of a B. pinnata was paced inside a custom-built branch enclosure chamber. b) An air pump, connected to the chamber via plastic tubing draws air out of the chamber, and through a sorbent tube where the VOCs emitted by the branch are collected. Later, sorbent tubes were run through GC-MS to determine VOCs emitted and to calculate ‘branch’ emission rate. Total above ground plant dry weight (g) was divided by branch dry weight and then multiplied by branch emission rate to give total plant emission rate (mg VOC / hour).

VOC samples were taken from a single *B. pinnata* branch (still attached to the main plant) inserted inside of a custom-built, 9 l branch enclosure (Extended Data Fig. 8). The two ends of the chamber were made from polytetrafluoroethylene supporting a transparent enclosure made from polyvinyl fluoride film (Dupont Chemicals). Ambient air, passed through an activated charcoal scrubber, was supplied to the chamber at 12 l min^−1^ using a mass flow controller (Aalborg). Supplementary photosynthetically active radiation (PAR) (380 μmol m^−2^ s^−1^) was provided by 20 W LED lights (Arlec). Mean air temperature inside the chamber was 23.3 °C. PAR and air temperatures inside the chamber were recorded automatically every minute using a Hobo H21 Micro Station Datalogger coupled with SLIAM003 PAR and S-THB-M002 temperature/relative humidity sensors (Onset). VOCs were collected from the enclosures using a sorbent tube containing 200 mg Tenax TA (Markes International) connected to an air pump (AirChek 2000; SKC) flowing air at 200 ml min^−1^ for 30 min. *B. pinnata* branches were allowed 15 min to acclimate to conditions within the enclosure before VOC sampling commenced. Background (control) enclosure samples were taken at the beginning and end of each day. Post-sampling tubes were maintained at 4 °C until analysis by GC-MS. Compounds were identified after GC-MS analysis using the same protocol used when developing *B. pinnata* odour profile.

Quantification of the compounds was made using the three major characteristic ions of the compounds in comparison to external standards diluted in methanol. All chemicals were purchased from Sigma-Aldrich. Emission rates (mg g(dw)^−1^ h^−1^) of each of the compounds were determined using their background subtracted concentrations, the chamber flow rate, sampling duration and the dry weight (dw) of the leaves of each branch. The mean emission rate per compound across all plants was summed and multiplied by the mean branch dry weight to determine the mean whole plant emission rate (mg h^−1^). Comparison with the vial emission rate showed that the whole plant emissions were on average 2.96 times greater than 1 ml vials. Hence, the volume of the compound mixtures was increased to 2.96 ml to give a comparable emission rate to the plants (Extended Data Table 1). Emissions of the informative VOCs from nursery plants were similar to those of the wild plants (Extended Data Fig. 2).

#### Virtual neighbour emission rate over time

Ten replicates of informative, flipped proportion and uninformative virtual neighbour vials were created to a volume of 2.96 ml, and the total weight of each vial was measured. All vials were placed on a heated plate under laboratory conditions (DBH20D dry block heater, Ratek Instruments) set at a constant 25 °C. Weights of vials were measured every 7 days and the slope of the weight loss over time determined to give an average emission rate (mg h^−1^).

#### Virtual neighbour odour dispenser

To deploy virtual neighbour vials at our study site, ensuring vials were secure and were not affected by rain, we created bespoke odour dispensers (Extended Data Fig. 4). Dispensers did not alter the VOC emission from virtual neighbours (analysis of similarity indicated no significant difference in VOCs emitted from virtual neighbour vials alone or when placed in dispensers, *R* = 0.092, *P* = 0.13, *n* = 10). To account for dispenser presence affecting wallaby foraging behaviour, we included a fourth ‘procedural control’ treatment comprising an *E. punctata* seedling surrounded by five evenly spaced dispensers with empty virtual neighbour vials.

#### Associational refuge main trial

All six treatments were deployed at our study site in plots (*n* = 15 per treatment, at least 50 m apart) in a completely randomized plot design. At each plot, five virtual or real neighbours were placed evenly in a circle (radius 1 m) around a single *E. punctata* seedling (325 ± 18 mm tall; Extended Data Fig. 5). All plants were sourced from Plants Plus Cumberland Forest Nursery and came potted (using Scott’s Osmocote Native Premium Potting Mix) in black plastic 200 mm ‘Garden City Plastic Grow Plant Pots’. Temperature ranged across the study period from 13.3 °C to 37.0 °C with a mean of 6.5 mm daily rainfall, with 12 days of rain (of >1 mm) over the total 40 day period (Terrey Hills, Sydney, Bureau of Meteorology 2022).

Patches were monitored for 40 days between February and March 2023 using motion-triggered infra-red trial cameras (ScoutGuard SG560K or SG2060-K; Professional Trapping Supplies). Cameras were fastened to wooden stakes (camera height = 0.7 m, distance to seedling = 1.5 m) at an approximate 45° angle towards the palatable seedlings. Cameras were set to record 60 s videos with instant re-trigger.

After 40 days, we quantified the survival time of palatable seedlings at ‘time to first wallaby browse (days)’ (when a wallaby consumed any part of the palatable seedling). If browsed, we estimated the percentage of foliage consumed from each of *E. punctata* seedlings as seen on camera using a visual estimate, with percentage intervals of 0%, 25%, 50%, 75% and 100% eaten.

#### Pre-trial period

Before the main trial, we ran a 14 day pre-trial period to both habituate wallabies to the experimental set-up of camera and stake and calculate a score of background wallaby activity per patch (background wallaby activity score = the number of wallabies recorded at a treatment site during the pre-trial period).

#### Statistical analysis

We used generalized linear models with a Poisson distribution and log link function in R (version 4.2.0; R Core Team, 2022, lme4 package^25^) to test whether there was a difference in background wallaby activity (dependent variable) between treatment sites.

We used Cox proportional-hazards models in R (‘survival’ package^26^) to model ‘survival’ (where failure is based on time to first browse) as a function of treatment and background wallaby activity (fixed factors). These models were also used to calculate pairwise hazard ratios between treatments. These analyses take into account right-censored data. Data were censored for any un-browsed seedlings by using the maximum number of hours until the end of the experimental period. We report the hazard ratio (exp(coef)) for all pairwise comparisons between treatments (Extended Data Table 2).

When significant differences existed, we performed Tukey post hoc tests to locate those differences (reported using alphabetical superscript in figures).

### Reporting summary

Further information on research design is available in the Nature Portfolio Reporting Summary linked to this article.

### Supplementary information

Supplementary Information Supplementary Note 1.

Reporting Summary

Peer Review File

Supplementary Data 1 Raw *B. pinnata* VOC peak area data collected across two sampling bouts.

## Figures and Tables

**Extended Data Table 1:**
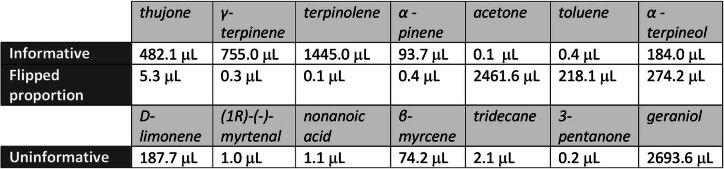
Compound volumes used in virtual neighbour treatments

Compound volumes used in virtual neighbour treatments, adjusted for exact compound concentration and volume to match real B. pinnata VOC paired proportions and emission rate. All virtual neighbour vials were mixed to a volume of 2.96 mL.

**Extended Data Table 2:**
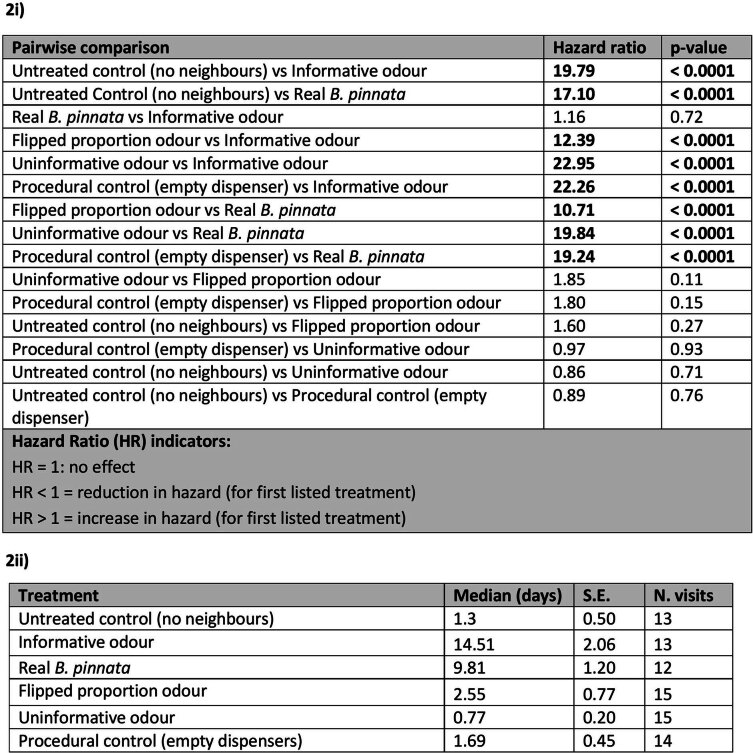
Virtual neighbourhood time to first wallaby browsed

**2i)** Virtual neighbourhood time to first wallaby browsed hazard ratios for pairwise comparisons between individual treatments. Significant treatment effect (Cox proportional-hazards model, LR χ_5^2 = 74.70, P < 0.0001). Hazard ratios indicate the likelihood that a treatment (first listed) will be browsed in comparison to another (second listed). High hazard ratios indicate a higher probability of being browsed. **2ii)** Of patches browsed, summary of median time to first wallaby browse in days, standard error (S.E) and number of browsing visits (N. visits).

## Data Availability

The datasets generated during and/or analysed during the current study are available in the Sydney eScholarship Repository^27^ (https://hdl.handle.net/2123/31657). Supplementary Data 1 provides a complete odour profile from odour headspace sampling undertaken.
