# Supplementary material for: Olfactory misinformation provides refuge to palatable plants from mammalian browsing
Source: Nat Ecol Evol. 2024 Feb 2;8(4):645–50. doi: 10.1038/s41559-024-02330-x (PMC11009103; doi:10.1038/s41559-024-02330-x)
Supplement: Supplementary file 1 — Supplementary Note 1. [file 41559_2024_2330_MOESM1_ESM.pdf]

nature ecology & evolution

Brief Communication https://doi.org/10.1038/s41559-024-02330-x

Olfactory misinformation provides refuge to palatable plants from mammalian browsing

In the format provided by the authors and unedited

Supplementary Note 1

Comparison of odour profiles between two wild *B.pinnata* odour sampling bouts.

B.pinnata odour headspace sampling (Supplementary Fig. 1) was undertaken across two sample bouts (March 2021, n = 10 and April 2022, n = 20). Our first sampling bout was undertaken on 26th March 2021 between 10:00 and 15:50. Ambient temperature ranged between 20.8°C – 24.3°C. From this sampling bout we identified 182 VOCs (Supplementary data matrix 1), 74 of which fell within our threshold for consistency (emitted by more than 50% of plants sampled). Our second sampling bout was undertaken on 2nd April 2022 between 09:00 and 16:00. Ambient temperature ranged between 19.5°C – 23.4°C. From this sampling bout we identified 393 VOCs (Supplementary data matrix 1), 55 of which fell within our threshold for consistency.

Combining both sampling bouts, the total odour profile for *B.pinnata* comprised of 485 VOCs. Of these 485, 90 VOCs overlapped between both sampling bouts, and 21 of the overlapping VOCs were consistent. The seven VOCs selected for informative virtual neighbours where among the 21 overlapping consistent VOCs between both bouts. A comparison of informative VOC pair proportions across both bouts can be seen in Supplementary Fig. 2.
