# Supplementary material for: Olfactory misinformation provides refuge to palatable plants from mammalian browsing
Source: Nat Ecol Evol. 2024 Feb 2;8(4):645–50. doi: 10.1038/s41559-024-02330-x (PMC11009103; doi:10.1038/s41559-024-02330-x)
Supplement: Supplementary file 2 — Reporting Summary [file 41559_2024_2330_MOESM2_ESM.pdf]

nature portfolio

Corresponding author(s): Patrick B. Finnerty
Last updated by author(s): Dec 11, 2023

Reporting Summary

Nature Portfolio wishes to improve the reproducibility of the work that we publish. This form provides structure for consistency and transparency in reporting. For further information on Nature Portfolio policies, see our Editorial Policies and the Editorial Policy Checklist.

Statistics

For all statistical analyses, confirm that the following items are present in the figure legend, table legend, main text, or Methods section.

n/a	Confirmed	
☐	☒	The exact sample size (*n*) for each experimental group/condition, given as a discrete number and unit of measurement
☐	☒	A statement on whether measurements were taken from distinct samples or whether the same sample was measured repeatedly
☐	☒	The statistical test(s) used AND whether they are one- or two-sided<br>*Only common tests should be described solely by name; describe more complex techniques in the Methods section.*
☐	☒	A description of all covariates tested
☐	☒	A description of any assumptions or corrections, such as tests of normality and adjustment for multiple comparisons
☐	☒	A full description of the statistical parameters including central tendency (e.g. means) or other basic estimates (e.g. regression coefficient) AND variation (e.g. standard deviation) or associated estimates of uncertainty (e.g. confidence intervals)
☐	☒	For null hypothesis testing, the test statistic (e.g. *F*, *t*, *r*) with confidence intervals, effect sizes, degrees of freedom and *P* value noted<br>*Give P values as exact values whenever suitable.*
☒	☐	For Bayesian analysis, information on the choice of priors and Markov chain Monte Carlo settings
☒	☐	For hierarchical and complex designs, identification of the appropriate level for tests and full reporting of outcomes
☒	☐	Estimates of effect sizes (e.g. Cohen's *d*, Pearson's *r*), indicating how they were calculated

Our web collection on statistics for biologists contains articles on many of the points above.

Software and code

Policy information about availability of computer code

Data collection	No software was used
Data analysis	We analysed the data collected in this study using R version 4.2.0.

For manuscripts utilizing custom algorithms or software that are central to the research but not yet described in published literature, software must be made available to editors and reviewers. We strongly encourage code deposition in a community repository (e.g. GitHub). See the Nature Portfolio guidelines for submitting code & software for further information.

Data

Policy information about availability of data

All manuscripts must include a data availability statement. This statement should provide the following information, where applicable:

- Accession codes, unique identifiers, or web links for publicly available datasets
- A description of any restrictions on data availability
- For clinical datasets or third party data, please ensure that the statement adheres to our policy

The datasets generated during and/or analysed during the current study are available in the Sydney eScholarship Repository, https://hdl.handle.net/2123/31657

Research involving human participants, their data, or biological material

Policy information about studies with human participants or human data. See also policy information about sex, gender (identity/presentation), and sexual orientation and race, ethnicity and racism.

Reporting on sex and gender	Not applicable
Reporting on race, ethnicity, or other socially relevant groupings	Not applicable
Population characteristics	Not applicable
Recruitment	Not applicable
Ethics oversight	Not applicable

Note that full information on the approval of the study protocol must also be provided in the manuscript.

Field-specific reporting

Please select the one below that is the best fit for your research. If you are not sure, read the appropriate sections before making your selection.

☐ Life sciences ☐ Behavioural & social sciences ☒ Ecological, evolutionary & environmental sciences

For a reference copy of the document with all sections, see nature.com/documents/nr-reporting-summary-flat.pdf

Ecological, evolutionary & environmental sciences study design

All studies must disclose on these points even when the disclosure is negative.

Study description

Stage 1 involved replicating the informative odour compounds of an avoided plant species, Boronia pinnata, to develop virtual neighbours. We sampled the odour 'headspace' of multiple real B. pinnata at our study site (n = 30) to develop a complete odour profile for this species. We then employed two 'Rules of reliability' to define the informative volatile organic compounds (VOCs) of B. pinnata. Identified informative VOCs (seven VOCs, into six pairs) were then mixed together into glass amber diffusion vials to form our informative virtual neighbour treatment. As a comparison, we combined the same number of VOCs (seven VOCs, into six pairs) that were recorded in B. pinnata but fell below our chosen reliability threshold as an uninformative virtual neighbour treatment. We inverted the relative amount of informative VOCs within pairs as a third flipped proportion virtual neighbour treatment. We then analysed the VOC emissions from these virtual neighbour vials (n = 10 per treatment, 30 total) and adjusted VOC volumes to ensure that VOCs were being emitted in the correct paired proportions identified from B. pinnata. Finally, we compared the VOC emission rate between informative virtual neighbour vials (n = 8) and real B. pinnata (n = 8), and adjusted VOC volumes to ensure that VOC emission rates were equivalent.

Stage 2 involved testing how swamp wallabies, Wallabia bicolor, responded to the three virtual neighbour treatments compared to real B. pinnata. This study was conducted on free-ranging wallabies in eucalypt woodland in eastern Australia. Virtual neighbours and real B. pinnata were deployed at our study site in plots (n = 15 per treatment, at least 50 m apart) in a completely randomised plot design. At each plot, five virtual or real neighbours were placed in a circle (radius 1 m) around a single highly palatable Eucalyptus punctata seedling at the centre of the plot. Virtual neighbour vials were deployed in bespoke virtual neighbour odour dispensers. As part of this experiment we also compared two additional treatments: a procedural control, and an untreated control (n = 15 per treatment). The untreated control treatment was a single E. punctata seedling. The procedural control treatment was a single E. punctata seedling surrounded by five empty virtual neighbour odour dispensers to ensure that any wallaby browsing effects were not due to the presence of the dispensers themselves.

Plots were monitored for 40 days between February and March 2023 using motion-triggered infra-red trail cameras. After 40 days, we quantified the survival time of E. punctata seedlings at 'time to first wallaby browse (days)' (when a wallaby consumed any part of the palatable seedling). If browsed, we quantified the proportion of E. punctata biomass consumed after the first browsing visit. We also recorded whether the palatable seedling was browsed during the day or during the night. Before this experiment, we ran a 14-day pre-trial period to both habituate wallabies to the experimental set-up of camera and stake and calculate a score of background wallaby activity per plot.

Research sample

Free-ranging swamp wallaby (Wallabia bicolor) population within Ku- ring- gai Chase National Park, Sydney, Australia (33°41′33″S, 151°08′44″E)

Sampling strategy

No sample size calculation was performed prior to the experiment, as the underlying variation in the data was not known. Our sample size was chosen based on previous work done on a similar scale (Bedoya-Pérez et al. 2014 Oecologia, Finnerty et al. 2017 J Anim Ecol, Orlando et al. 2020 Bio Letters)

Data collection

Stage 1: Data was collected both from the field, and in the lab by two observers. Stage 2: Data was collected from footage collected

Data collection	by motion-triggered infra-red trail cameras placed directly in the field by one observer.
Timing and spatial scale	Stage 1: B.pinnata odour sampling was undertaken across two sampling bouts (March 2021, n = 10 and April 2022, n = 20). Randomly selected individual plants sampled were of approximate equal height (198 ± 11 cm) and were at least 50 m away from any other sampled individual. Development of virtual neighbours was completed between April 2022 - February 2023.<br><br>Stage 2: Treatments were deployed and camera-trapping occurred between February 2023 - March 2023. Treatment plots and cameras were spaced at least 50 m apart.
Data exclusions	No data was excluded from our analysis.
Reproducibility	No measures have been taken to verify the reproducibility of the experimental findings.
Randomization	All six treatments were deployed at our study site in plots in a completely randomised plot design.
Blinding	Complete blinding was not possible as treatments could be partially determined from camera trap footage. However, as virtual neighbour vials were deployed in blackened odour dispensers, informative, uninformative, flipped proportion, and procedural control treatments could not be distinguished from one another. There was also little to no room for interpretation as data collection was a simple recording of when a wallaby first browsed on a seedling within a plot, how much of the seedling was consumed, and for how long.

Did the study involve field work? ☒ Yes ☐ No

Field work, collection and transport

Field conditions	Stage 1: B. pinnata odour sampling at our study site was conducted cross two bouts in March 2021 and April 2022, between 8 am to 5 pm respectively. Ambient temperatures recorded were similar across both bouts (March 2021: 20.8°C – 24.3°C, April 2022: 19.5°C – 23.4°C) and average daily rainfall was slightly higher in March 2021 (14.3 ± 4.7 mm) than in April 2022 (7.8 ± 3.0 mm).<br><br>Stage 2: Between February and March 2023 temperature at our study site ranged from 13.3 °C to 37.0 °C with a mean of 6.5 mm daily rainfall, with 12 days of rain (of > 1 mm) over the total 40-day period .
Location	Our study site was adjacent to Murrua Trail, Murrua Side Trail, and Gibberagong Trail within Ku- ring- gai Chase National Park, Sydney, Australia (33°41′33″S, 151°08′44″E)
Access & import/export	Research was conducted under a NSW Government Department of Planning, Industry and Environment Scientific Licence (Biodiversity Conservation Act 2016), Licence number: SL102186.
Disturbance	There was no disturbance caused by the study.

Reporting for specific materials, systems and methods

We require information from authors about some types of materials, experimental systems and methods used in many studies. Here, indicate whether each material, system or method listed is relevant to your study. If you are not sure if a list item applies to your research, read the appropriate section before selecting a response.

Materials & experimental systems

n/a	Involved in the study	
☒	☐	Antibodies
☒	☐	Eukaryotic cell lines
☒	☐	Palaeontology and archaeology
☐	☒	Animals and other organisms
☒	☐	Clinical data
☒	☐	Dual use research of concern
☒	☐	Plants

Methods

n/a	Involved in the study	
☒	☐	ChIP-seq
☒	☐	Flow cytometry
☒	☐	MRI-based neuroimaging

Animals and other research organisms

Policy information about studies involving animals; ARRIVE guidelines recommended for reporting animal research, and Sex and Gender in Research

Laboratory animals	The study did not involve laboratory animals.

Wild animals	Swamp wallabies (Wallabia bicolor) were observed using non-invasive motion-triggered infra-red trail cameras on plots. The animals were not captured or handled in any way. The age of wild swamp wallabies observed could not be determined.
Reporting on sex	This information was not collected.
Field-collected samples	The study did not involve samples collected from the field.
Ethics oversight	Animal ethics approval was granted by the University of Sydney's Animal Ethics Committee (protocol number 2022/2196).

Note that full information on the approval of the study protocol must also be provided in the manuscript.
